# Linking enzyme sequence to function using conserved property difference locator to identify and annotate positions likely to control specific functionality

**DOI:** 10.1186/1471-2105-6-284

**Published:** 2005-11-30

**Authors:** Kimberly M Mayer, Sean R McCorkle, John Shanklin

**Affiliations:** 1 Biology Department, Brookhaven National Laboratory, Upton, NY 11973, USA

## Abstract

**Background:**

Families of homologous enzymes evolved from common progenitors. The availability of multiple sequences representing each activity presents an opportunity for extracting information specifying the functionality of individual homologs. We present a straightforward method for the identification of residues likely to determine class specific functionality in which multiple sequence alignments are converted to an annotated graphical form by the Conserved Property Difference Locator (CPDL) program.

**Results:**

Three test cases, each comprised of two groups of funtionally-distinct homologs, are presented. Of the test cases, one is a membrane and two are soluble enzyme families. The desaturase/hydroxylase data was used to design and test the CPDL algorithm because a comparative sequence approach had been successfully applied to manipulate the specificity of these enzymes. The other two cases, ATP/GTP cyclases, and MurD/MurE synthases were chosen because they are well characterized structurally and biochemically. For the desaturase/hydroxylase enzymes, the ATP/GTP cyclases and the MurD/MurE synthases, groups of 8 (of ~400), 4 (of ~150) and 10 (of >400) residues, respectively, of interest were identified that contain empirically defined specificity determining positions.

**Conclusion:**

CPDL consistently identifies positions near enzyme active sites that include those predicted from structural and/or biochemical studies to be important for specificity and/or function. This suggests that CPDL will have broad utility for the identification of potential class determining residues based on multiple sequence analysis of groups of homologous proteins. Because the method is sequence, rather than structure, based it is equally well suited for designing structure-function experiments to investigate membrane and soluble proteins.

## Background

Useful functional information can be extracted from amino acid sequences using a comparative strategy to identify potential SDPs (specificity determining positions) that differ between functionally divergent homologous proteins that arose from a common ancestor. In such an alignment, amino acids at positions important for a particular function are expected to be well-conserved within, but different between, the functional classes. The identification of potential SDPs not only deepens our understanding of the relationship of amino acid sequence to protein function but such knowledge can also be put to practical use. For example, several protein engineering groups have used the comparative strategy to alter substrate specificity (for example [1-3]) and Broun *et al*. [4] used a comparative strategy to successfully engineer an enzyme to convert its function (desaturation) to that of a divergent homolog (hydroxylation) and *vice-versa*.

Multiple sequence alignments of homologous amino acid sequences used in structure-function studies are often compared manually. The process typically involves iterative rounds of sequence alignment in which sequences are added or removed and the effects of doing so are evaluated. Such comparisons tend to be labor intensive, error prone, and become impracticable as the number of sequences in the data set increases. Furthermore, the number and complexity of comparisons grow rapidly with the increasing amount of protein sequence data available in public databases. Yet this growing data resource contains a wealth of information for structure-function studies and for protein engineering. We recognized a need for a general tool for extracting and displaying relevant functional information from such data sets.

**Figure 1:**
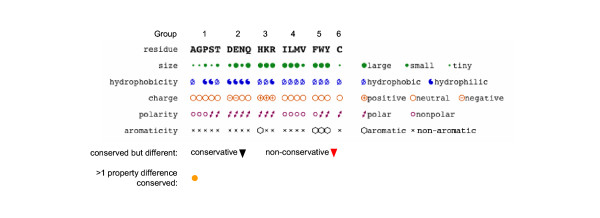
The CPDL key. Amino acid residues are arranged such that changes within each of the six groups are conservative and changes between groups are non-conservative.

**Figure 2:**
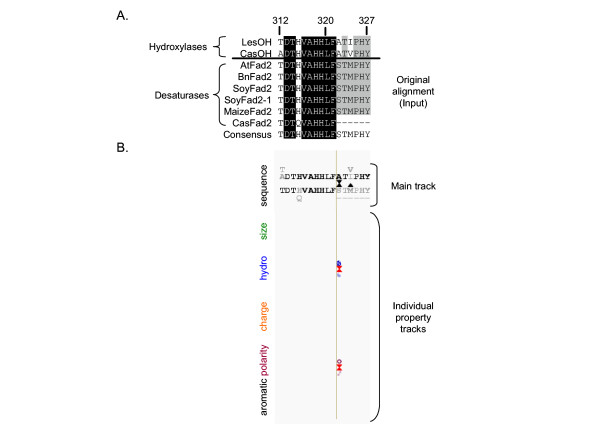
(A) A portion of the alignment of two plant fatty acid hydroxylases and seven desaturases (residues 312–327 of *Arabidopsis *Fad2). (B) An example of CPDL output for the same portion of the alignment. Positions with conserved amino acid residues differences are marked with triangles in the main track. This display was generated using the mask that flags residue properties only for those positions that are marked with triangles in the main track. For example, position 322 has hydrophobic, nonpolar alanine in class 1 but is hydrophilic, polar serine in class 2. The CasFad2 enzyme sequence from castor bean is incomplete.

To extract this relevant functional information, we have developed the Conserved Property Difference Locator (CPDL) program to compare the alignment of two classes of homologous proteins and then identify and flag those positions where the consensus property differs between the classes. Properties include amino acid sequence as well as size, hydrophobicity, charge, polarity and aromaticity (Fig. 1). In the case of amino acid sequence, the program also distinguishes between conservative and non-conservative substitutions. The inclusion of descriptive properties of the amino acids makes it easy to visualize those positions where amino acid sequence is not conserved within a class but other properties are conserved but different from the properties found at the equivalent position in the other class (Fig. 2). The CPDL program provides a way to evaluate large sequence comparisons and identify only those positions most likely to control class-specific functions.

A number of automated methods based on functional sequence conservation have been designed to address a related problem, namely predicting sub-types in order to categorize sequences (protein) into the correct subfamilies and functional classes. [5-11]. Many of these programs require multiple inputs such as a 3D structure as well as many sequences per class, and some provide an annotated multiple sequence alignment in addition to several pages of text that must be interpreted together with the alignment to be of value (for example, the AMAS program [5]).

We developed the 3D structure-independent program CPDL as a way to simplify and extract only that information pertinent to the identification of potential SDPs between two homologous enzyme classes and to display the output as an easily interpretable graphic in which all the information is displayed as a series of tracks alongside a contiguous consensus sequence for each of two homologous classes.

## Implementation

### CPDL program description

CPDL was implemented in mzScheme [12] on a Unix system along with two custom-written Perl-script multiple sequence alignment format converters on the front end (.msf and .aln currently). To facilitate ease of use, a web interface was created for CPDL [13]. At the entry form, the user uploads the prepared multiple sequence alignment, enters the row number which divides the two protein classes, sets the gray-scale preferences and chooses property-masking levels in the display status section. The graphic output is rendered as a PDF or Postscript output and is sent to the user's browser which can be configured to auto-launch an appropriate viewer such as xpdf. [14], Acrobat Reader. [15], Ghostscript, or Ghostview. [16].

### Organization of CPDL input

CPDL evaluates an amino acid alignment which includes proteins of two classes, each consisting of at least two members per class (CPDL is not an alignment tool). Use of the CPDL program first requires the creation of a suitable amino acid alignment of all the proteins of interest using a program like ClustalX [17], T-Coffee [18], or Dialign [19]. The construction of an accurate alignment is a prerequisite for CPDL input but selection of an appropriate program (for review see [20]) must be empirically determined for each data set. Manual adjustments to the alignment or consideration of the 3D structure for the alignment, while not required, may improve the quality of the CPDL input for some data sets. The single alignment used as CPDL input must be formatted so that the sequences of all members of class 1 occupy rows 1 through N and all members of class 2 fall below row N. The multiple sequence alignment used as input may contain a large number of sequences, limited only by the total size of the sequence alignment file (currently arbitrarily set at 1 MB to maintain speed of the webserver). A file of 1 MB would approximately correspond to an alignment containing 800 sequences of 400 amino acids each.

### Description of CPDL output

CPDL produces a graphical output consisting of a set of horizontal tracks with positions corresponding to the input alignment (see Fig. 2 for an example of CPDL input and output). The upper portion of the output contains the main track which shows the amino acid residues present in each class of sequences as a consensus sequence. The first consensus is of the class 1 sequences and displays all amino acids found at each position, with the most frequent in the class on the main track and the remainder listed above in order of decreasing frequency. The second consensus is of the class 2 sequences and is displayed the same way, except that additional residues are stacked below the main track in order of decreasing frequency (Fig. 2). To further aid in quickly identifying conserved positions, the relative frequency of the amino acid is indicated by gray-scale, the most frequently occurring being the darkest. Thus, completely-conserved positions appear as single dark amino acids, less conserved positions appear lighter and more dispersed from the main track (Fig. 2).

The lower portion of the output contains the "individual property tracks" which are constructed similarly to the main sequence track but display consensus of different residue properties (size, hydrophobicity, charge, polarity and aromaticity). The properties for each amino acid are defined as in Taylor [21]. Symbols are used to indicate properties (Fig. 1) and they are also arranged with the most frequently occurring printed the darkest followed by less common properties in rank order above (in class 1) or below (in class 2).

The user may define the level of conservation such that either "all" or "all-but-one" residues within a class must match (except when there are only two sequences in a class, in which case both must match to be conserved). The all-but-one designation is intended to mitigate the effect of sequencing errors that might be present in the source data. Conserved positions are likewise defined for residue properties.

If a residue (or property) is conserved in one class and different from the most common residue at the same position in the other class, a triangle is placed in the CPDL output between the consensus sequences, pointing toward the other class. Thus, positions where each class has a conserved but different residue (or property) are flagged with a double triangle (hourglass). If the conserved residue from class 1 is not found at that position in any of the members of class 2, the triangle is filled. However, if there is at least one member with the same residue at that position in class 2, the triangle remains open. The triangles are colored black if the change is conservative (Fig. 1) or red if the change is non-conservative [22]. Finally, an orange circle is placed in the main track at those positions which do not show a conserved residue difference but where there is at least one residue property difference that is conserved.

We have established a masking hierarchy that can be selected by the user directing CPDL to describe (i) every property at every position, or (ii) flag all positions that have any change in sequence or property and list the properties, or (iii) flag only those positions that are conserved in sequence and list their properties if different, or finally (iv) flag those positions with conserved, non-conservative amino acid changes and list their properties if different.

## Results

We evaluated the ability of CPDL to identify residues whose properties are conserved within classes but that differ between classes using three test data sets. Each set represents a large enzyme family with clearly-defined subtypes for which experimental data regarding functional residues is available, allowing us to assess the CPDL output in terms of its ability to identify residues that contribute to functional identity. In addition, structural data is available for test cases 2 and 3, allowing us to interpret the CPDL output in its structural context. For the purposes of the following comparisons, we define potential SDPs as those where each class has a conserved residue that differs from the conserved residue found in the other class at a given position (flagged by two filled triangles). For other experimental data sets, this definition may be modified as desired by the user to include amino acid positions where only one class has a conserved residue, or where the residues are not conserved but the amino acid properties are conserved.

### Test case 1: Fatty acid desaturases and hydroxylases

**Figure 3:**
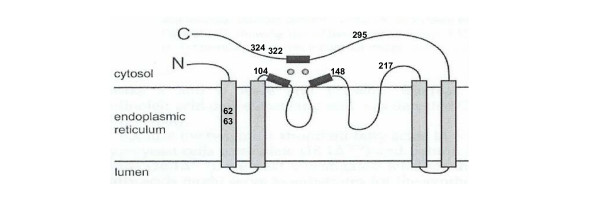
The location of CPDL-identified potential SDPs shown on a topological model of the membrane-bound Fad2 enzyme (based on [28, 29]). Position 62 is conserved in the hydroxylase class and different in the desaturases; all other positions are conserved in the desaturases and different in the hydroxylases (residues are listed in Table 1).

Desaturase members of the Fad2 family of enzymes introduce double bonds at the 12-position in a fatty acid chain, while hydroxylase members of the family instead introduce an -OH group at the same position of the same substrate. [4,23]. Still other members act as acetylenases [24,25], conjugases [26] or epoxidases [24,27] at the same position. While a crystal structure remains to be determined for any membrane-bound desaturase, a topological model of the enzyme has been proposed [28,29] (Fig. 3).

In a standard multiple sequence alignment of two hydroxylases with six desaturases (28% identity overall), Broun *et al*. [4] identified seven positions out of ~390 that exhibited strict conservation among the desaturases but were different from residues in equivalent positions in the hydroxylases. Two positions contain non-conservative residue changes (T148I/N and A295V; numbered according to Fad2 with the desaturase residues listed first), the other five contain conservative changes (A63S/V, A104G, Y217F, S322A, and M324I/V). CPDL flagged the same seven positions previously identified (Fig. 3) in addition to one position not reported by Broun *et al*. [4] (I/F62L; Table 1 and Fig. 3).

**Table 1:** CPDL-identified positions in the desaturase/hydroxylase family. The amino acid residues present in each class are listed, with the most common residue given first as applicable.

Position	Hydroxylase	Desaturase
62	L	I/F
63	S/V	A
104	G	A
148	I/N	T
217	F	Y
295	V	A
322	A	S
324	I/V	M

Broun *et al*. [4] substituted the seven conserved residues found in the desaturases for their equivalents in a hydroxylase and *vice versa*. The result was that a desaturase was converted into a hydroxylase and a hydroxylase into a desaturase. Further experiments showed four of the seven positions are principally responsible for the change in functionality [4]. Broadwater *et al*. [30] subsequently showed that positions 148 and 324 exert the greatest influence on functional outcome in terms of desaturation versus hydroxylation. This example shows that CPDL identified a set of eight positions, seven identified previously by Broun *et al*. [4] and an additional one, and that positions identified in this way include the two found to principally control the functional outcome. This example also demonstrates that CPDL analysis can be useful in the absence 3D structural information.

### Test case 2: ATP/GTP cyclases

Tucker [3] used a homology modeling approach of the GTP cyclase on the crystallographically-determined ATP cyclase structure to identify potential SDPs. The nucleotidyl cyclase family of enzymes converts nucleotide triphosphates to cyclic nucleotide monophosphates that can activate kinases and regulate ion channels. Their strict substrate specificity is important for proper physiological function (reviewed in [31]).

From ~150 positions in the catalytic domains of the nucleotidyl cyclases, Tucker [3] identified five potential SDPs (K938E, Q1016R, D1018C, I1019L, and W1020F, numbered according to the ATP cyclase PDB id 1AB8, with the ATP cyclase residues listed first). Only two of these amino acid substitutions (K938E and D1018C) were required to convert the function of a GTP cyclase to an ATP cyclase [3].

**Figure 4:**
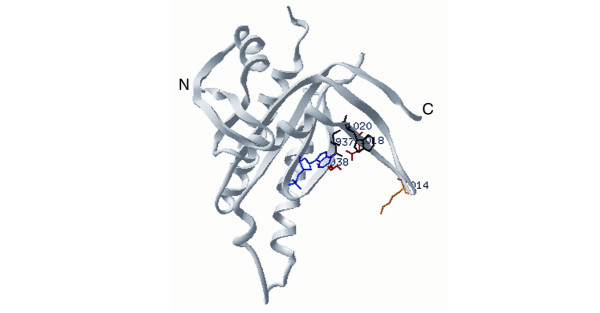
The location of CPDL-identified potential SDPs in the ATP cyclase structure (PDB id 1CUL). Only positions that are conserved within a group and different between groups are shown. Red residues are positions in ATP cyclase that have a non-conservative substitution in GTP cyclase; black residues indicate positions at which conservative substitutions are located (residues are listed in Table 2). The substrate (adenosine triphosphate) is shown in blue.

We subjected a multiple sequence alignment comprised of the catalytic domains of 20 ATP cyclases and 16 GTP cyclases to CPDL for evaluation and identified four potential SDPs (Table 2 and Fig. 4). These four positions include the two non-conservative substitutions shown by Tucker [3] to convert specificity (K938E and D1018C) in addition to two positions with conservative substitutions (I937V and W1020F). Thus CPDL was able to identify four residues (from ~150) that potentially affect function, two of which were previously identified by a structure-based approach and shown to be critical for functional determination [3].

**Table 2:** CPDL-identified positions in the nucleotidyl cyclase family. The amino acid residues present in each class are listed.

Position	GTP Cyclase	ATP Cyclase
937	V	I
938	E	K
1018	C	D
1020	F	W

### Test case 3: Mur synthetases

The ATP-dependent UDP-N-acetylmuramoyl-L-alanine:D-glutamate (MurD) and UDP-N-acetylmuramoyl-L-alanyl-D-glutamate:*meso*-diaminopimelate (MurE) ligases catalyze consecutive steps in the prokaryotic peptidoglycan pathway. MurD and MurE recognize different UDP-sugar and amino acid substrates, however, they are similar in amino acid sequence and 3D structure and are hypothesized to employ the same catalytic mechanism[32]. The enzymes of the peptidoglycan biosynthetic pathway are under intense study as targets for antibacterial therapeutics because the pathway is essential for viability (for review, see [33]). Manual comparisons of their amino acid sequences have been used to identify potential active site residues [34].

Structures of MurD bound to substrate (UDP-Mur-NAc-L-Alanine), product (UDP-Mur-NAc-L-Alanine-D-Glutamate), and adenosine 5'-diphosphate [35, 36] allowed us to evaluate the CPDL program output in a structural context [34]. For CPDL evaluation, we chose 20 unique MurD and 25 unique MurE sequences from among the highest-identity sequences to the biochemically-defined archetypes of MurD and MurE.

**Figure 5:**
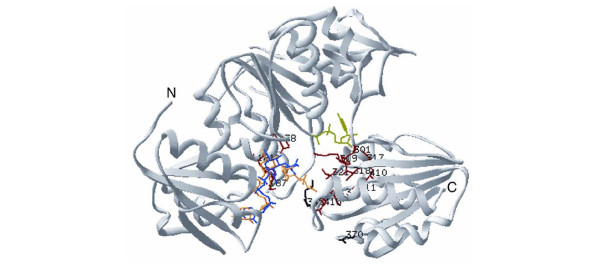
The location of CPDL-identified potential SDPs in the MurD structure (PDB id 3UAG). The MurD substrate UDP-Mur-NAc-L-Alanine is shown in blue, the product UDP-Mur-NAc-L-Alanine-D-Glutamate in orange, adenosine 5'-diphosphate in green. Only positions that are conserved within a group and different between groups are shown. Red residues are positions in MurD that have a non-conservative substitution in MurE; black residues indicate positions at which conservative substitutions are located (residues are listed in Table 3).

From >400 positions in the MurD/E sequences, CPDL identified a total of 14 positions where the MurD sequence was conserved (all or all-but-one) but different from the conserved MurE sequence at the same position (Table 3). Of these 14 potential SDPs, ten residue differences were non-conservative (N138T, F161H, Y187H, H301G, DSK317-319VDY, T321H, C413G, and S415E; numbered according to MurD with the MurD residue listed first) while four were conservative (K348R, G370T, SP410-411AG). Examination of these positions in the crystal structures revealed that all of the positions are located in the active site cleft (Fig. 5) and each is near one of three experimentally-determined binding sites [35, 36]. Specifically, residues 317–319, 321, 348, 370, 410–411, 413, and 415 are all near the D-glutamate binding site in MurD [36]. The equivalent region of MurE binds diaminopimelic acid and thus differences between the homologs would be expected in this region. Differences would also be expected in the C-terminal domain since the MurE product (UDP-Mur-NAc-Tripeptide) is longer by two pentapeptide chains and thus extends deeper into the C-terminal domain than does the MurD substrate (UDP-Mur-NAc-L-Alanine).

**Table 3:** CPDL-identified positions in the Mur synthetase family. The amino acid residues present in each class are listed, with the most common residue given first as applicable.

Position	MurD	MurE
138	N	T
161	F	H
187	Y	H
301	H	G
317	D	V/I
318	S	D
319	K	Y/F
321	T	H
413	C	G
415	S	E

CPDL identifies potential SDPs in the same region as earlier manual sequence comparisons. However, although both 194 and 198 were previously implicated as potential SDPs [34], CPDL does not identify these positions because they are conserved across both classes. Recent experiments show a functional requirement for a K at position 198 in MurD, MurE, and MurF. [37]. Additionally, CPDL discounted position 425 that was previously proposed as an SDP [34] because it is not highly conserved in MurE sequences.

## Discussion

We present an analysis of three test cases in which CPDL identified sets of positions constituting a small fraction of the total amino acid sequence that included experimentally validated SDPs. The positions primarily responsible for defining class-specific functions between the desaturase and hydroxylase members of the Fad2-like family of enzymes. [4,30] were identified. The potential SDPs that CPDL flagged for the nucleotidyl cyclases contain two residues previously identified by a structure-based approach and shown experimentally to be important determinants of specificity [3]. Our results with the MurD/E ligase family demonstrate that CPDL-identified potential SDPs are primarily located in regions of the proteins shown experimentally and/or predicted to be important for function and/or specificity. Taken together, the results from these three independent test cases suggest that CPDL-identified positions are likely to be contained within the enzyme active site, providing a link between amino acid sequence, structure, and enzyme function. These positions can thus serve as starting points for detailed structure-function studies. The fact that CPDL analysis for all three test cases, including one integral membrane and two globular enzyme families, yielded a small number of amino acid positions that included those reported to contribute to specificity suggests CPDL will be generally useful for analysis of other families of enzymes.

One property of CPDL that contributes to its utility is the graphic output comprised of a pair of consensus sequences with potential SDPs marked with flags. The output is directly comparable to the input multiple sequence alignment which is useful for visualizing whether the potential SDPs fall within regions of otherwise high homology that are likely to represent active sites. Furthermore, CPDL has the ability to display each of the properties (size, hydrophobicity, charge, polarity, and aromaticity) as well as sequence for every residue in a protein alignment, making it possible to distinguish between potential SDPs based on property conservation (e.g. D/E changes are flagged differently than K/E changes). CPDL also incorporates a user-defined masking hierarchy allowing for the optimization of the output for each comparison. We note that CPDL allows for the identification of potential SDPs without a requirement for a 3D structure, a feature that makes it suitable for the study of membrane proteins for which there are few crystal structures available.

CPDL is unique in that it uses a distinct flag for those positions where one class has a conserved residue but where at least one member of the other class contains the same residue (open triangles). Because CPDL is heavily dependent on the quality of the multiple sequence alignment, users are advised to evaluate the input data with great care. Accurate CPDL output is also dependent on correct functional classification. Thus, in cases where several open triangles are attributable to the same input sequence, it may be desirable to either exclude the sequence from analysis or confirm its classification experimentally. CPDL also identifies positions where properties other than sequence (e.g. charge or hydrophobicity) are conserved within classes but differ between classes. These positions may also represent specificity-determining positions and so may warrant experimental testing.

The CPDL program is also well suited for fine mapping of chimeric enzymes that have been constructed to coarsely map specificity-determining regions of an enzyme. Furthermore, since the CPDL input alignment is user defined portions of interest within proteins e.g., domains can be evaluated separately.

## Conclusion

We developed the CPDL to identify residue positions that affect specificity and/or functionality and tested the program using one integral membrane, and two soluble globular, enzyme families. The results obtained from CPDL analysis were consistent with available biochemical and structural data regarding specificity-determining positions of these enzymes, suggesting this program will be of broad utility in assisting the design of structure-function studies on other enzyme families.

## Availability and requirements

• **Project name: **Conserved Property Difference Locator (CPDL)

• **Project home page: **

• **Operating system(s): **Platform independent

• **Programming language: **Perl, mzScheme

• **Other requirements: **A standard HTML web browser, PDF display program such as Acrobat reader or xpdf, or a postscript display program such as ghostscript.

• **License: **GNU GPL

• **Any restrictions to use by non-academics: **Covered by GNU GPL license

## Authors' contributions

JS and KMM conceived the study. SRR implemented the algorithm and developed the software and web-interface. KMM and SRR tested the software. KMM collected and evaluated the test data sets. KMM, SRR and JS drafted the manuscript. All authors have read and approved the manuscript.
